# Arctigenin Efficiently Enhanced Sedentary Mice Treadmill Endurance

**DOI:** 10.1371/journal.pone.0024224

**Published:** 2011-08-26

**Authors:** Xuan Tang, Jingjing Zhuang, Jing Chen, Liang Yu, Lihong Hu, Hualiang Jiang, Xu Shen

**Affiliations:** 1 State Key Laboratory of Drug Research, Shanghai Institute of Materia Medica, Chinese Academy of Sciences, Shanghai, China; 2 School of Pharmacy, East China University of Science and Technology, Shanghai, China; Paris Institute of Technology for Life, Food and Environmental Sciences, France

## Abstract

Physical inactivity is considered as one of the potential risk factors for the development of type 2 diabetes and other metabolic diseases, while endurance exercise training could enhance fat oxidation that is associated with insulin sensitivity improvement in obesity. AMP-activated protein kinase (AMPK) as an energy sensor plays pivotal roles in the regulation of energy homeostasis, and its activation could improve glucose uptake, promote mitochondrial biogenesis and increase glycolysis. Recent research has even suggested that AMPK activation contributed to endurance enhancement without exercise. Here we report that the natural product arctigenin from the traditional herb *Arctium lappa* L. (Compositae) strongly increased AMPK phosphorylation and subsequently up-regulated its downstream pathway in both H9C2 and C2C12 cells. It was discovered that arctigenin phosphorylated AMPK via calmodulin-dependent protein kinase kinase (CaMKK) and serine/threonine kinase 11(LKB1)-dependent pathways. Mice treadmill based in vivo assay further indicated that administration of arctigenin improved efficiently mice endurance as reflected by the increased fatigue time and distance, and potently enhanced mitochondrial biogenesis and fatty acid oxidation (FAO) related genes expression in muscle tissues. Our results thus suggested that arctigenin might be used as a potential lead compound for the discovery of the agents with mimic exercise training effects to treat metabolic diseases.

## Introduction

Physical inactivity is considered as one of the risk factors for the development of type 2 diabetes and other metabolic diseases. It is known that endurance exercise training could lead to fiber type transformation, mitochondrial biogenesis, angiogenesis and other adaptive changes in skeletal muscle [1], [2], thus further enhancing fat oxidation that is associated with improvement of insulin sensitivity in obesity [3]. Currently, at least 60% of the global population fails to achieve the daily minimum recommendation of 30 min moderate intensity of physical activity, and within these people the risk of getting the related chronic diseases including type 2 diabetes increases by 1.5 times [4]. Therefore, it has become valuable to discover active agents that would mimic the effects of exercise training to prevent or treat metabolic diseases.

AMP-activated protein kinase (AMPK) is a heterotrimeric serine/threonine protein kinase with three subunits (α, β, γ) [5]. As the major molecular sensor for AMP/ATP ratio in cells, AMPK plays a pivotal role in the regulation of energy metabolism [6]. AMPK activation switches on ATP-producing processes (such as glucose uptake, mitochondrial biogenesis and glycolysis) and inhibits ATP-consuming anabolic processes (such as protein synthesis and sterol synthesis) [7]. AMPK phosphorylation is regulated by a series of upstream AMPK kinases, including serine/threonine kinase (LKB1) [8], Tak1 kinase and two calmodulin-dependent protein kinase kinases (CaMKKα and CaMKKβ) [9], [10].

Recently, it was reported that AMPK activation could improve mice endurance in the absence of exercise training [11]. Under endurance training condition, skeletal muscle suffers a number of changes, such as glucose consumption decreasing, main energy source transition from glucose to fatty acid utilization, mitochondrial biogenesis increasing and fiber-type switch [11], [12]. AMPK activation could increase peroxisome proliferator-activated receptor-γ coactivator-1α (PGC-1α) gene expression or the direct phosphorylation of PGC-1α in skeletal muscle [6], [13]. PGC-1α is known to be involved in the regulation of mitochondrial biogenesis, respiration, hepatic gluconeogenesis and other biological processes by interaction with several transcription factors, such as ERRα, NRF1, NRF2 and PPARs [14], [15]. PGC-1α null mice showed muscle dysfunction, abnormal weight control and hepatic steatosis [16], [17]. Although skeletal muscle was not an active lipogenic organ as liver, exercise training or AMPK activation has been also reported to promote fatty acid synthesis and oxidation as evidenced by up-regulation of varied main enzymes such as pyruvate dehydrogenase kinase 4 (PDK4), stearoyl-CoA desaturase-1 (SCD-1), fatty acid synthetase (FAS) and muscle carnitine palmitoyltransferase I (mCPT1b) within related pathways in skeletal muscles [18]–[20]. Oxidation of fatty acid exports more energy than metabolism of glucose, and alteration of utilizing this energy substrate could contribute to exercise tolerance [11], [21]. Additionally, AMPK activation changes skeletal muscle myofiber type composition, which mimics the fiber type switch induced by endurance training [11], [22]. Therefore, all the above findings suggested that AMPK might act as a key mediator of endurance training-induced changes, which was also confirmed further by the result that treatment of AMPK agonist AICAR at a dose of 500 mg/kg/day could induce metabolic genes expression and enhance running endurance [11].

**Figure 1:**
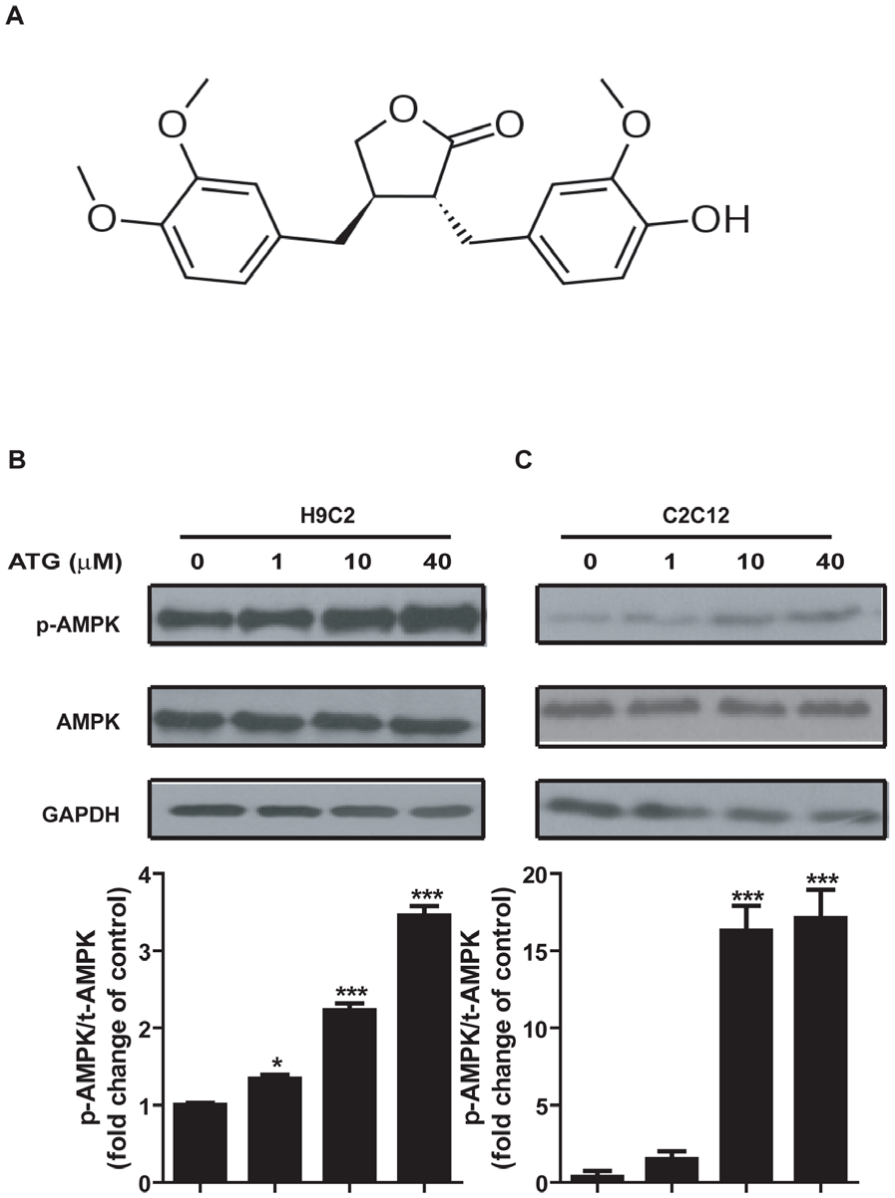
Arctigenin (ATG) increased AMPK phosphorylation in H9C2 and C2C12 cells. **A**. Chemical structure of arctigenin. **B**. **C** H9C2 (B) and differentiated C2C12 (C) cells were treated with indicated concentrations of arctigenin (0-40 µM) for 30 min, AMPK phosphorylation and total AMPK levels were determined by western blotting. The results shown are representative of three independent experiments. The bands were quantified using Image-Pro Plus software. Values are means ± SE. *, p<0.05; ***, p<0.005; one-way ANOVA.

Arctigenin (ATG, Fig. 1A) is a phenylpropanoid dibenzylbutyrolactone lignan extracted from the traditional herb *Arctium lappa* L. (Compositae) with anti-cancer and anti-inflammatory effects [23]–[26]. As a new type of antitumor agent, arctigenin could block the unfolded protein response (UPR) by reducing the expression of UPR-related genes, such as C/EBP homologous protein (CHOP), activating transcription factor 4 (ATF4) and glucose-regulated protein of 78 kDa (GRP78) under glucose deprivation [23]–[25]. Its anti-inflammatory effect mainly acts by inhibiting type I-IV allergic inflammation and pro-inflammatory enzymes *in vitro* and *in vivo* [26].

In the present study, we discovered that arctigenin could increase AMPK phosphorylation and up-regulate its downstream-pathway related genes mRNA levels to promote mitochondrial biogenesis and fatty acid synthesis and oxidation *in vitro* and in *vivo*, subsequently leading to the mice treadmill endurance enhancement. Cell based assay revealed that arctigenin increased AMPK phosphorylation targeting the CaMKK and LKB1-dependent pathways. Our results thus demonstrated that arctigenin might be used as a lead compound for the discovery of the agents with mimic exercise training effects to treat metabolic diseases.

## Results

### Arctigenin increased AMPK phosphorylation in H9C2 and C2C12 cells

As reported, AMPK activation switched on ATP-producing processes to enhance endurance [7], [11]. With this information, we thus constructed the phospho-AMPK activator screening platform (Text S1), based on which our lab in-house natural product library was screened out and the natural product arctigenin (Fig. 1A) was finally identified to efficiently activate AMPK phosphorylation (Fig. S1). As shown in Fig. 1B and C, arctigenin dose-dependently increased AMPK phosphorylation in both H9C2 and C2C12 muscle cells, while had no effects on total AMPK.

### Arctigenin activated PGC-1α transcription via up-regulating AMPK phosphorylation

**Figure 2:**
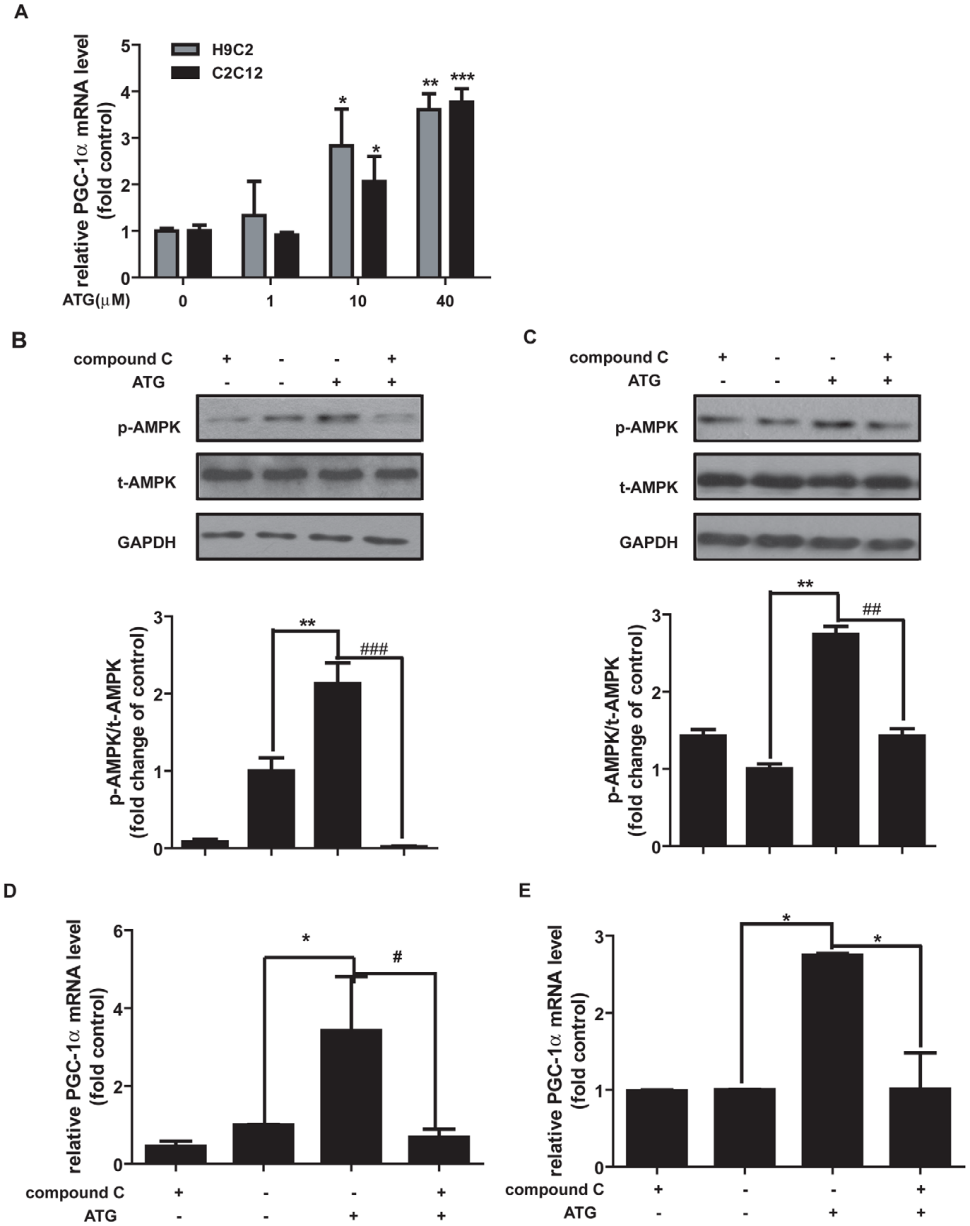
Arctigenin (ATG) increased PGC-1α transcription via enhancing AMPK phosphrylation. **A**. H9C2 cells and differentiated C2C12 cells were cultured with arctigenin or DMSO for 24 hours before harvest. Total RNA extraction, cDNA preparation and PGC-1α mRNA quantification were performed as “Experimental Procedures”. GAPDH mRNA was used as an internal control and data was shown as folded changes of blank control. **B**. **C**. H9C2 cells (B) and differentiated C2C12 cells (C) were treated with or without 20 µM compound C for 1 hour before and during the incubation with actigenin (20 µm) for 24 hours. After harvest, phospho- and total AMPK protein levels were analyzed. The bands were quantified using Image-Pro Plus software. Values are means ± SE. **D**. **E**. H9C2 (D) and differentiated C2C12 cells (E) were treated with or without 20 µM compound C for 1 hour before and during the incubation with actigenin (20 µM) for 24 hours. Total mRNA was extracted and PGC-1α mRNA level quantified. The results shown are representative of three independent experiments. *, p<0.05; **, p<0.01; ***, p<0.005; one-way ANOVA. #, p<0.05; ###, p<0.005: for compound C and arctigenin co-incubation group versus arctigenin treated group; student's t test.

PGC-1α was known to act as the master regulator in mitochondrial biogenesis and skeletal muscle adaptation [27]–[29]. Since AMPK activation by actual exercise or pharmacological treatment could lead to up-regulation of PGC-1α gene expression [30], [31], we investigated the potential effects of arctigenin on PGC-1α mRNA level in both H9C2 and C2C12 cells. Compared with DMSO-treated group, arctigenin incubation could dose-dependently up-regulate PGC-1α mRNA levels in both cell lines (Fig. 2A). By considering that AMPK phosphorylated PGC-1α directly at theronine-177 and serine-538, which are required for induction of PGC-1α promoter [6], we thus examined whether arctigenin could increase PGC-1α transcription through regulating its promoter activity by luciferase assay. As shown in Fig. S2A, the relative PGC-1α promoter activity was highly increased in arctigenin treated group compared with the DMSO group in HEK293T cells.

PGC-1α as a key regulator in energy metabolism could respond to varied physical stimuli, such as muscle contraction, cold stress and overfeeding [32]–[35]. It could be regulated by AMPK, p38 MAPK or NF-κB pathway [11], [36]–[38]. Since arctigenin has been determined to increase AMPK phosphorylation and PGC-1α transcription, we thus wondered whether the effect of arctigenin on PGC-1α was dependent on its effect on AMPK. Therefore, we examined the effects of arctigenin on PGC-1α mRNA level and PGC-1α promoter activity together with AMPK inhibitor compound C incubation in the related cells. As shown in Fig. 2B–E and Fig. S2B, treatment of compound C almost inhibited the arctigenin-induced AMPK phosphorylation and completely blocked the arctigenin-induced up-regulation of PGC-1α mRNA level and promoter activity, implying that the effect of arctigenin on PGC-1α transcription regulation was dependent on its role in AMPK phosphorylation.

These results thereby indicated that arctigenin could induce PGC-1α transcription in skeletal muscle and cardiac muscle cell lines via up-regulating AMPK phosphorylation.

### Arctigenin increased mitochondrial biogenesis and fatty acid oxidation genes expression

**Figure 3:**
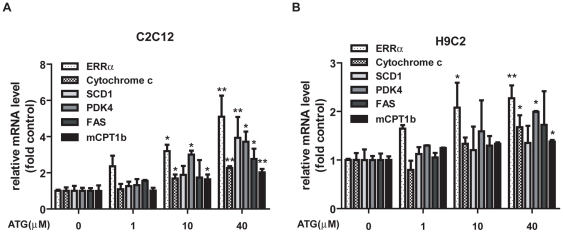
Arctigenin (ATG) promoted mitochondrial biogenesis and FAO related gene expression. **A**. **B**. H9C2 and differentiated C2C12 cells were treated with indicated concentration of arctigenin (1, 10, 40 µM) or DMSO for 24 hours before harvest. Total RNA extraction, cDNA preparation and ERRα, cytochrome c, SCD1, PDK4, FAS, and mCPT1b mRNA quantification were performed as “Experimental Procedures”. GAPDH RNA was used as an internal control for calculating mRNA fold changes. The results shown are validated by three independent experiments. *, p<0.05; **, p<0.01; ***, p<0.005; one-way ANOVA.

Since arctigenin has been determined to induce PGC-1α transcription via up-regulating AMPK phosphorylation, this result thereby implied that arctigenin might play a potential role in promoting mitochondrial biogenesis and function. To further evaluate this hypothesis, the mRNA levels of the related genes were detected in both H9C2 and C2C12 cell lines with results listed in Fig. 3A and B. Estrogen-related receptor α (ERRα), a nuclear receptor activated by interacting with PGC-1α, was reported to control the expression of nuclear genes encoding mitochondrial proteins (NUGEMPs) [39], [40]. We found ERRα mRNA level was obviously elevated by arctigenin treatment. At the same time, as a typical NUGEMP and significant component of electron transport chain (ETC) in mitochondrial, cytochrome c mRNA was also induced by arctigenin administration. Furthermore, as also indicated in Fig. 3A, arctigenin significantly activated the mRNA levels of PDK4, SCD1, FAS and mCPT1b which are key enzymes in fatty acid synthesis and oxidation for promotion of energy source transforming from glucose to fatty acid in C2C12 cells at 40 µM, while the regulation of arctigenin in these genes was not effectively in H9C2 cells for lack of significant difference in SCD1 and FAS mRNA levels between high dose of arctigenin administration group (40 µM) and DMSO control (Fig. 3B).

To clarify whether arctigenin regulated mitochondrial biogenesis and FAO related genes mRNA levels through its effect on AMPK phosphorylation, we examined the influence of arctigenin on these genes together with AMPK inhibitor (compound C) incubation in C2C12 and H9C2 cells. As indicated in Fig. S3 and S4, treatment of compound C almost inhibited the arctigenin-induced genes expression except mCPT1b in H9C2 and SCD1 in C2C12 cells. These results thus demonstrated that arctigenin increased mitochondrial biogenesis and fatty acid oxidation genes mRNA levels mainly through its effect on AMPK phosphorylation.

Therefore, all our results suggested that arctigenin could activate PGC-1α transcription and increase mitochondrial biogenesis and fatty acid oxidation genes expression in both skeletal muscle and cardiac muscle cells.

### Arctigenin regulated the related genes expression not via affecting transcriptional activity of PPARδ

As reported, AMPK catalytic subunit over-expression evidently promoted basal and ligand-dependent transcription of PPARδ [11], indicative of that PPARδ might be also involved in AMPK related gene expression. The fact that AICAR (AMPK activator) synergistically increased mice endurance and gene expression with GW501516 (PPARδ agonist) further implied the potential of AMPK-PPARδ signaling axis. Here, we also examined whether arctigenin could induce the related PPARδ involved gene expression with mammalian one-hybrid and transcriptional activation assay systems. As shown in Fig. S5, arctigenin failed to regulate the co-activator recruitment or transcriptional activity of exogenous or endogenous PPARδ.

Therefore, all above-mentioned results suggested that arctigenin regulating the relative gene expression is related to the enhancement of AMPK phosphorylation, while might not to the promotion of PPARδ transcriptional activity.

### Arctigenin enhanced AMPK phosphorylation through CaMKK and LKB1-dependent pathways

**Figure 4:**
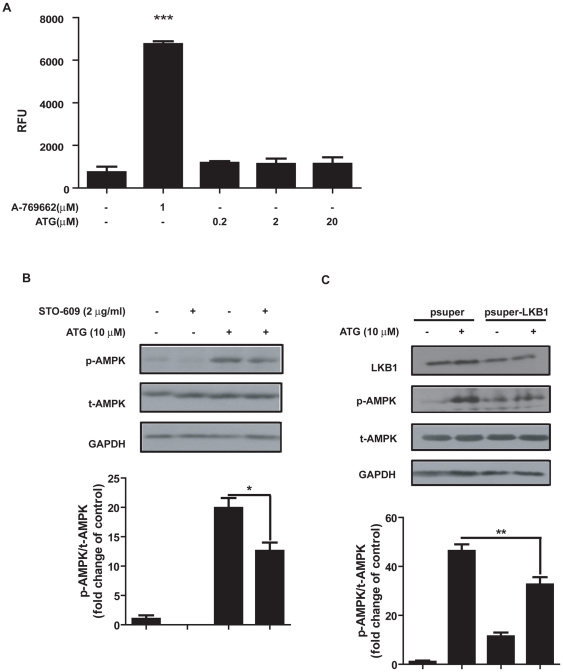
Arctigenin (ATG) enhanced AMPK phosphorylation through CaMKK and LKB1-dependent pathway. **A**. Arctigenin was pre-incubated with AMPK α2β1γ1 for 30 min, AMPK activity was measured by monitoring the produced ADP with A-769662 as a positive control. **B**. HEK293T cells were treated with arctigenin (10 µM) in the absence or presence of STO-609 (2 µg/ml, pre-incubation for 4 h in serum-free DMEM) for 30 min in serum-free DMEM. AMPK phosphorylation and total AMPK levels were determined by western blotting. **C.** After transfected with pSuper.neo.gfp-LKB1 for 48 hours, HEK293T cells were treated with arctigenin (20 µM) for 30 min. AMPK phosphorylation and total AMPK levels were determined by Western blotting. The bands were quantified using Image-Pro Plus software. Values are means ± SE. Values are means ± SE. The results shown are representative of three independent experiments. *, p<0.05; **, p<0.01; ***, p<0.005; student's t test.

Considering that arctigenin could activate AMPK phosphorylation, we subsequently investigated the potential regulation of this natural product against the relevant AMPK-involved pathways. As determined, directly activating AMPK in an allosteric manner and/or indirectly promoting Thr172 phosphorylation of AMPK both contributed to the AMPK activation. Bear that in mind, we examined the recombinant AMPK enzyme activity with or without arctigenin incubation *in vitro* to investigate whether arctigenin could influence the conformation of AMPK thus inducing AMPK activity. As indicated in Fig. 4A, arctigenin had no effect on the recombinant AMPK enzyme activity, while AMPK agonist A-769662 as a positive control significantly increased the phosphorylation of its substrate SAMS [41], [42], implying that arctigenin was not an AMPK ligand and indirectly activated AMPK.

As reported, AMPK has an obligate requirement for phosphorylation by an upstream kinase on Thr-172 in the α-subunit catalytic domain [5]. Since the above assay has indicated that arctigenin activated AMPK in an indirect manner, we further explored the potential signaling responsible for arctigenin-induced AMPK activation. Regarding the fact that LKB1 and CaMKK are within the main identified kinases in AMPK upstream pathway and play pivotal roles in regulation of AMPK phosphorylation [43], we thus addressed these two kinases related assays. Firstly, we determined whether CaMKK activation was necessary for arctigenin-induced AMPK phosphorylation. As shown in Fig. 4B, pretreatment of HEK293T, a model cell usually used in mechanism studies, with the selective CaMKK inhibitor STO-609 [9] obviously attenuated the arctigenin-induced AMPK phosphorylation. To investigate whether LKB1 pathway might participate in the arctigenin-induced AMPK activation, we carried out LKB1 knock-down assay in HEK293T cells as reported [44]. The results in Fig. 4C revealed that LKB1 knock-down in HEK293T cells efficiently down-regulated the arctigenin-induced AMPK phosphorylation.

Taken together, our results thereby suggested that arctigenin stimulated AMPK phosphorylation via CaMKK and LKB1-dependent pathways.

### Arctigenin efficiently enhanced sedentary mice treadmill endurance

**Figure 5:**
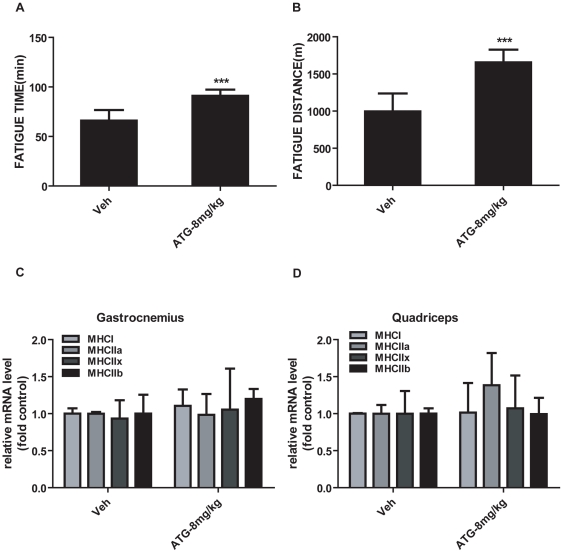
Arctigenin (ATG) elevated mice treadmill performance without inducing myofiber type conversion in skeletal muscle. A. B. After mice were administrated with arctigenin (8 mg/kg) or vehicle via intraperitoneal for a period of 6 weeks, they were run to fatigue on a treadmill as “Experimental Procedures”. Running time and distance were recorded (n = 8/group). Values are means ± SD. C. D. Total RNA was extracted from gastrocnemius and quadriceps, and type I and type II MHCs were then analyzed by real-time PCR assays (n = 10/group). GAPDH RNA was used as an internal control for calculating mRNA fold changes. ***, p<0.005; student's t test.

AMPK was reported as an “exercise mimetic”, whose pharmacological activator-AICAR was ever tested to provide several benefits of exercise in sedentary mice [11]. Since arctigenin has been determined able to effectively activate AMPK phosphorylation, we thereby evaluated whether this natural product could enhance mice endurance. To address this issue, we performed the treadmill exhaustion test among the selected mice (All mice were examined regarding the treadmill performance before arctigenin administration, and those mice whose running time was too long or too short compared with the average were eliminated to reduce the potential effects by the inherent variation) after 6-week arctigenin administration (8 mg/kg). Running time and distance till fatigue were estimated as maximal endurance capacity. As shown in Fig. 5A and B, arctigenin administration brought on approximately an increase of 40% in mean fatigue time and 65% in mean fatigue distance, further indicating that arctigenin efficiently enhanced sedentary mice treadmill endurance.

During the treatment, mice showed similar basal behavior, food consumption and body weight (Fig. S6A and B). In addition, to investigate the preliminary toxicity of arctigenin, TNFα and IL-6 levels in mice serum were tested. The results revealed that there was no difference in those two inflammatory factors comparing arctigenin with vehicle groups (Fig. S6C and D). At the same time, aspartate aminotransferase (AST) and alanine aminotransferase (ALT) were also detected. As shown in Fig. S6E and F, no change was found for AST while ALT elevated with arctigenin administration, suggestive of the potential hepatotoxicity of arctigenin in *ip* administration. Therefore, these results demonstrated that arctigenin elevated sedentary mice endurance efficiently while no significant toxicity was observed.

### Arctigenin could not induce skeletal muscle fiber-type changes

As reported, endurance exercise, AMPK mutation with persistent activation and PGC-1α over-expression in transgenic mice driven by muscle creatine kinase (MCK) promoter could induce myofiber type switch [12], [22], [45]. We thereby considered that arctigenin might change myofiber type construction in skeletal muscle tissues. However, we failed to find any obvious conversions in the mRNA levels of four different myosin heavy chain (MHC) isoforms (MHCI as a marker to represent type I myofibers, MHCIIa, MHCIIx and MHCIIb as markers to represent type II myofibers) in gastrocnemius (Fig. 5C) and quadriceps (Fig. 5D) comparing arctigenin administration group with vehicle group. Additionally, no obvious changes were found in myofibers compositions between arctigenin treatment group and vehicle group in ATPase staining (Fig. S7). These results thus suggested that arctigenin could not affect myofiber type proportion.

### Arctigenin induced AMPK phosphorylation, mitochondrial biogenesis and FAO pathway in vivo

**Figure 6:**
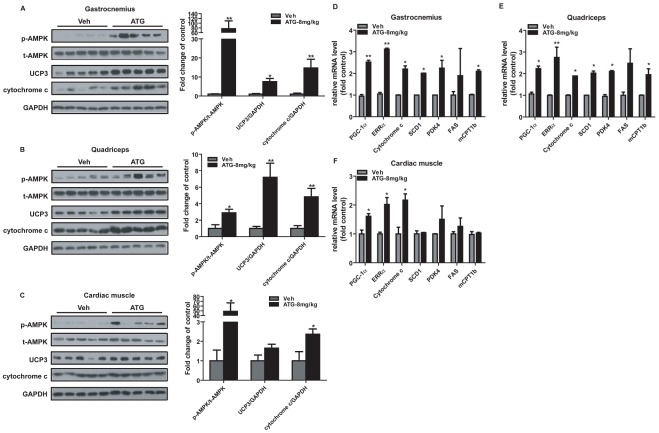
Arctigenin (ATG) enhanced AMPK phosphorylation, mitochondrial biogenesis and FAO pathway in vivo. **A**. **B**. **C**. Protein levels of p-AMPK, t-AMPK, UCP3 and cytochrome c were determined by western blotting in gastrocnemius (A), quadriceps (B) and cardiac muscle (C). (n = 5/group). The bands were quantified using Image-Pro Plus software. Values are means ± SE. **D**. **E**. **F**. Relative gene mRNA levels (PGC-1α, ERRα, cytochrome c, SCD1, mCPT1, PDK4, FAS) from gastrocnemius (D), quadriceps (E) and cardiac muscle (F) were analyzed by real-time PCR assays (n = 10/group). GAPDH RNA was used as an internal control for calculating mRNA fold changes. *, p<0.05; **, p<0.01; student's t test.

To further investigate the potential regulative mechanism of arctigenin regarding its improvement of mice treadmill endurance, we addressed the relevant tissue-based assays. Compared with vehicle group, arctigenin (8 mg/kg) administration enhanced AMPK phosphorylation in gastrocnemius (Fig. 6A), quadriceps (Fig. 6B) and cardiac muscles (Fig. 6C). The results shown in Fig. 6D–F suggested that arctigenin up-regulated the mRNA levels of PGC-1α and ERRα and the protein levels of cytochrome c in gastrocnemius, quadriceps and cardiac muscles. Additionally, mRNA levels of SCD1, PDK4 and mCPT1b were also determined to be obviously elevated in gastrocnemius and quadriceps muscles with arctigenin treatment, consistent with the cell based results. Notably, there was also a tendency for higher average FAS mRNA levels in arctigenin treated group but without significance. To further identify the regulation of arctigenin on FAO pathway, the protein level of uncoupling protein 3 (UCP3) that is also involved in FAO induction [46] was examined. As shown in Fig. 6A–C, UCP3 expression was obviously elevated in gastrocnemius and quadriceps muscles.

As fatty acid synthesis and storage related genes were up-regulated, we thereby hypothesized that arctigenin treated mice might contain more fatty acid in skeletal muscle for instant oxidation to supply energy source during exercise. Bearing that in mind, we thus detected fatty acid levels in skeletal muscles (gastrocnemius and quadriceps) and found that arctigenin treatment could enhance fatty acid storage in gastrocnemius evidently while in quadriceps without significance (Fig. S8A and B).

Therefore, all those *in vitro* and *in vivo* results thereby suggested that arctigenin could enhance AMPK phosphorylation, mitochondrial biogenesis and FAO pathway related genes expression, finally leading to the enhancement of exercise-free endurance.

## Discussion

Over the past decades, aerobic endurance exercise has been potently highlighted concerning its significance in the clinical amelioration of many disease symptoms, such as glucose metabolism in type 2 diabetes [47], dyslipidemia in atherosclerosis [48] and hypertension in stroke, acute myocardial infarction or cardiac insufficiency [49], [50]. Nevertheless, the inability to afford definite intensity of physical exercise has been always the obstacle to make profits of exercises [51]. Discovery of active agents that would mimic the reprogramming metabolism induced by exercise training is one of the effective strategies to overcome these obstacles.

Actual exercises could result in activation of kinases/phoshatases signaling pathway, nuclear translocation of transcription/translation factors, up-regulation of NUGEMPs and mtDNA-encoded proteins, and augmentation of muscle aerobic capacity [52]. AMPK is activated during physical activities to promote down-stream metabolic reprogramming (e.g. mitochondria biogenesis, fatty acid oxidation promotion and myofiber type switch), and considered as a significant mediator in skeletal muscle adaptations [6], [7], [22]. Therefore, pharmacological activation of AMPK may provide benefits of endurance exercise without actual physical activities, which was ever confirmed by endurance enhancing activity of its activators AICAR [11]. It was found that AMPK activator-AICAR, which is metabolized to an AMP mimetic in cell, could increase sedentary mice treadmill endurance at a high dosage of 500 mg/kg/day via intraperitoneal injection and up-regulate genes linked to oxidation metabolism via AMPK-PPARδ signaling axis [11]. Resveratrol, a natural polyphenolic product derived from grapes, could also increase mice aerobic capacity without exercise at a dose of 400 mg/kg/day orally [53] targeting SIRT1 and subsequently regulating its downstream pathway, which was reported to tightly couple with AMPK [54].

Compared with synthetic compounds, small molecules from natural sources are featured by their large-scale of structure diversity [55]. Therefore, we performed the screening of the efficient phosphor-AMPK activator targeting our in-house natural product library and finally discovered that arctigenin dose-dependently increased AMPK phosphorylation *in vitro* (Fig. 1B and C) and *in vivo* (Fig. 6A–C).

Arctigenin was extracted from *Arctium lappa* L., which has been widely used in traditional Chinese medicine [56]. Previous studies have illustrated that arctigenin was active in anti-viral infection, anti-tumor, anti-inflammation, and neuroprotection [23]–[26], [57]–[59]. Here we reported that arctigenin promoted AMPK phosphorylation on Thr172 site through CaMKK and LKB1-dependent pathways (Fig. 4). Recently, AMPK Ser485/491 phosphorylation was also reported involving in regulation of AMPK activity [60], [61]. To clarify whether arctigenin could affect AMPK phosphorylation on Ser485/491, we examined Ser485/491 phosphorylation levels of AMPK in arctigenin treated cells. As shown in Fig. S9, arctigenin did not change AMPK Ser485/491 phosphorylation in HEK293T, H9C2 or C2C12 cell lines, implying that arctigenin activated AMPK phosphorylation on Thr172 without impacting AMPK Ser485/491 phosphorylation.

We also found that arctigenin significantly enhanced sedentary mice running endurance at a dose of 8 mg/kg/day (Fig. 5A and B). Furthermore, cytochrome c protein and mRNA levels were typically increased in mitochondrial biogenesis, and FAO related genes (SCD1, PDK4, FAS and mCPT1b) mRNA levels were also obviously elevated in muscle tissues (Fig. 6). Fatigue induced by treadmill running was primarily developed from periphery tissues and featured by rapid clearance of intracellular ATP and relative insufficiency of oxidation metabolism in cardiovascular and skeletal muscle system [62]. We thus concluded that promotion of mitochondrial biogenesis and FAO linked gene expression induced by arctigenin attributed to mice prolonged running time and distance.

Myofiber type switch induced by endurance training was ever considered as one of the reasons for exercise tolerance [12], [22], [45], but we could not find any differences of fiber type composition in skeletal muscle tissues (gastrocnemius and quadriceps) between arctigenin-treatment and vehicle groups (Fig. 5C–D and Fig. S5). Compared with AMPK persistent activating mutation that led to myofiber type switch *in vivo* [45], the indirect phosphorylation of AMPK by arctigenin might only share part of the down-stream genes response with AMPK persistent activating mutation, which resulted in unchangeable myofiber type composition in arctigenin administrated group. Meanwhile, PGC-1α was also reported playing a pivotal role in myofiber type transformation via activating calcineurin signaling pathway [22]. Although arctigenin obviously up-regulated PGC-1α transcription *in vitro* (Fig. 2A and B) and *in vivo* (Fig. 6D–F), it might not mobilize calcium/calcineurin pathway or totally activate PGC-1α interaction with related proteins for promotion of the myofiber type switch. It is noted that arctigenin up-regulated mitochondrial biogenesis related genes (such as ERRα and cytochrome c) both in cardiac and skeletal muscle tissues, but elevated FAO related genes only in skeletal muscle tissues, which might be tentatively attributed to the tissue specificity responding to arctigenin in fatty acid metabolism.

**Figure 7:**
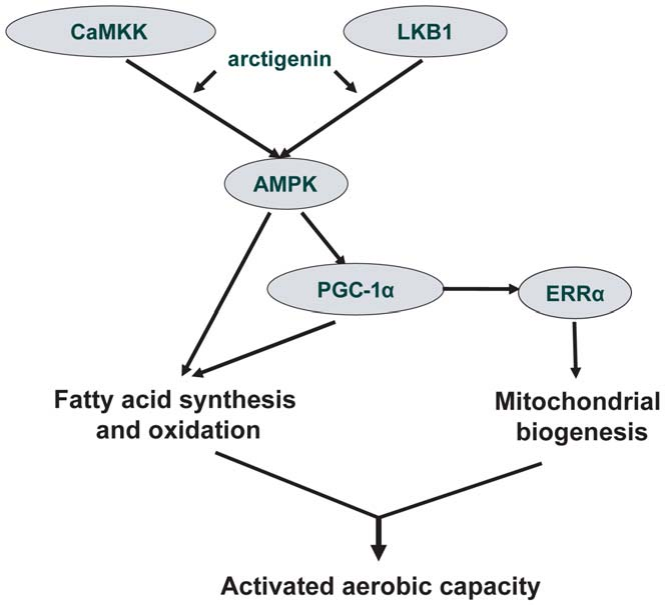
A proposed model demonstrating arctigenin (ATG)-induced endurance enhancement mechanism. Arctigenin induced AMPK phosphorylation via CaMKK and LKB1 pathways, resulting in PGC-1α up-regulation. Phosphorylated AMPK and PGC-1α activated fatty acid synthesis and oxidation. Meanwhile, PGC-1α co-activated ERRα to promote mitochondrial biogenesis. Promotion of mitochondrial biogenesis and FAO led to activated aerobic capacity.

In summary, we demonstrated that arctigenin could efficiently increase rodent sedentary treadmill endurance via enhancing AMPK phosphorylation. This natural product induced the accommodation of mitochondrial biogenesis and FAO pathway to promote mitochondrial oxidative capacity without actual physical activities as summarized in Fig. 7. Our results have provided additional understanding of pharmacological functions for arctigenin and traditional Chinese medicine *Arctium lappa* L., and suggested the potential of arctigenin as a lead compound for anti-chronic metabolic disease (e.g. obesity or type 2 diabetes) drug discovery.

## Materials and Methods

### Ethics Statement

All animal experiments were carried out in accordance with the Regulations of Experiments Animal Administration issued by the State Committee of Science and Technology of the People's Republic of China. Permit numbers: SCXK (HU) 2008-0017; SYXK (HU) 2008-0049. This study was approved by Science and Technology Commission of Shanghai Municipality.

### Materials

Restriction enzymes were purchased from New England Biolabs. Cell culture plastic ware was purchased from Corning Inc. DMEM, fetal bovine and horse serums were purchased from Invitrogen. Compound C and STO609 were obtained from Sigma. Calcium Phosphate Cell Transfection Kit was obtained from Beyotime. RNAiso, RT reagent Kit and SYRB Premix Ex Taq were purchased from TaKaRa. Dual Luciferase Assay System was obtained from Promega. Anti-cytochrome c, anti-phospho-AMPK (Thr172), anti-AMPKα1/α2, and anti-LKB1 antibodies were purchased from Cell Signaling Technology. Anti-CaMKK antibody was purchased from Senta Cruz Biotechnology. HEK293T, H9C2 and C2C12 cells were obtained from ATCC.

### Cell culture and differentiation

As a typical cardiac muscle cell line, H9C2 was derived from embryonic BD1X rat heart tissue and not differentiated in our study. C2C12 was a subclone of the mouse myoblast cell line and differentiated in DMEM with 2% horse serum, forming contractile myotubes and expressing characteristic muscle proteins. Differentiated myotubes were used in our experiments.

H9C2 and C2C12 cell lines were maintained in DMEM supplemented with 10% fetal bovine serum and the cells were grown at 37°C in an environment of 5% CO_2_. To induce myoblast fusion and myotubes differentiation, C2C12 myoblasts were switched to differentiation medium when 100% confluent in 6-well plate. Differentiation medium was exchanged every 2 days for 6 days before experimental manipulation.

### Western Blot analysis

Tissues were lysed with lysis buffer containing 25 mmol/L Tris-HCl (PH 7.5), 150 mmol/L NaCl, 1 mmol/L Na_3_VO_4_, 1% Triton X-100 and a protease inhibitor cocktail (Sigma-Aldrich). Protein concentrations were determined using a BCA protein assay kit (Pierce, Rockford, IL). Equal amounts of lysates or supernatants of the whole cell extracts were fractionated by SDS-PAGE and transferred to Hybond-c nitrocellulose membrane (Amersham Bioscience). The membranes were blocked for one hour at room temperature and then incubated overnight at 4°C in TBST buffer (5% milk) containing related antibody. The membranes were then incubated for an hour at room temperature in TBST buffer (5% milk) containing anti-rabbit IgG or anti-mouse IgG (Jackson-ImmunoResearch, West Grove, PA). Blots were visualized by incubation with SuperSignal West Dura chemiluminescence kit (Pierce Biotechnology) and exposing to light-sensitive film.

The bands were quantified as “intensity×area” using Image-Pro Plis software (MediaCybernetics) and statistically analyzed. SE was calculated from three repeats of the experiments or five individuals of arctigenin treated and vehicle groups.

### RT-PCR and quantitative real-time PCR

Total RNA was extracted from administrated cells or mice tissues using RNAiso (TaKaRa) reagent in accordance with the Kit instruction. cDNA was synthesized by RT reagent Kit (TaKaRa) and real-time PCR was performed using SYRB Premix Ex Taq (TaKaRa) on DNA Engene Opticon TM2 system (MJ Research, Waltham, MA, USA). The primers were listed as follows:

PGC-1α: sense 5′- gcccggtacagtgagtgttc-3′, anti 5′- ctgggccgtttagtcttcct-3′.

ERRα: sense 5′- ctcagctctctacccaaacgc-3′, anti 5′- ccgcttggtgatctcacactc-3′.

Cytochrome c: sense 5′- cagcttccattgcggacac-3′, anti 5′- ggcactcacggcagaatgaa-3′.

PDK4: sense 5′- agggaggtcgagctgttctc-3′, anti 5′- ggagtgttcactaagcggtca-3′.

FAS: sense 5′- ggaggtggtgatagccggtat-3′, anti 5′- tgggtaatccatagagcccag-3′.

mCPT1b: sense 5′- tgggactggtcgattgcatc-3′, anti 5′- tcagggtttgtcggaagagaga-3′.

SCD1: sense 5′- ttcttgcgatacactctggtgc-3′, anti 5′- cgggattgaatgttcttgtcgt-3′.

GAPDH: sense 5′- acagcaacagggtggtggac-3′, anti 5′- tttgagggtgcagcgaactt-3.

MHCI: sense 5′- ccttggcaccaatgtcccggctc-3′, anti 5′- gaagcgcaatgcagatgcggtg-3.

MHCIIa: sense 5′- atgagctccgacgccgag-3′, anti 5′- tctgttagcatgaactggtaggcg-3.

MHCIIx: sense 5′- aaggagcaggacaccagcgccca-3′, anti 5′- atctctttggtcactttcctgct-3.

MHCIIb: sense 5′- gtgatttctcctgtcacctctc-3′, anti 5′- ggaggaccgcaagaacgtgctga-3.

### Luciferase assay

For evaluation of the effects of arctigenin on PGC-1α promoter, HEK293T cells (24-well plate) were transfected with pGL3-PGC-1α-luc together with PRL-SV40 and refreshed with normal medium 5 hours later. After transfection, HEK293T cells were incubated with indicated concentration of arctigenin for 24 h. Luciferase activity was measured using Dual Luciferase Assay kit.

### AMPK enzymatic activity assay

The recombinant AMPK activity was assayed using the modified conventional approach in non-radioactive way (such as Jun N-terminal kinase 2 (Jnk2α2), casein kinase 1, Protein kinase A (PKA), etc.) [63], as outlined in Fig. S10.

In the assay, the substrate SAMS was used according to the literature methods [41], [42]. The recombinant AMPK isoform α2β1γ1 was purchased from Invitrogen. AMPK was diluted to 400 ng/ml in assay buffer (pH 7.4, containing 15 mM HEPES, 20 mM NaCl, 1 mM EGTA, 0.02% Tween) and pre-incubated with arctigenin (0.2, 2, 20 µM) or A-769662 (1 µM) for 30 min on ice. The kinase reaction was initiated by adding ATP (50 µM) and SAMS (50 µM) at room temperature for 30 min. The produced ADP reflecting the AMPK enzyme activity was thus measured by the ADP Hunter Plus Assay kit (DiscoverX), and the fluorescent signal was detected with an M5 Multi-Detection Reader using excitation and emission wavelengths of 530 and 590 nm.

### Animal experiment

C57BL/6J male mice at 6 weeks of age were purchased from Shanghai Experimental Animal Center, Chinese Academy of Sciences, and acclimated to SPF microisollators for 2 days before any experimental intervention. Mice were accommodated under standard conditions (strict 12:12-h light-dark cycle, 22°C, 60% humidity) in plastic cages and provided with water and food ad libitum.

All 30 mice were adapted to treadmill running for 10 min at 10 m/min at week -1 (5 days/week) avoiding unexpected accidents and the first fatigue test was conducted at week 0. For fatigue test, mice ran at 10 m/min for 5 min and 15 m/min for 10 min. After the initial warm-up period, exercise intensity was increased by 5 m/min every 30 min from 20 m/min until mice could not be prompted to continue running by moderate electric stimulation (less than 0.1 milliampere) and stayed at electrode for at least 10 sec. After the first fatigue test, 20 mice with moderate endurance capacity were selected from total 30 mice and divided into arctigenin administration and vehicle treatment groups (n = 10/group). Before final fatigue assay, sedentary mice were treated with arctigenin (8 mg/kg, body weight/day) or vehicle (sterilized 0.9% Sodium Chloride containing 5% Tween-80) daily via intraperitoneal injection for 6 weeks. Mice endurance capacity was estimated by treadmill (Litai Science and Technology Inc. Exer6, Hangzhou, China) running time and distance until fatigue [11], [62]. After 6-week arctigenin administration, the last fatigue test was performed according to the same protocol as before (n = 8/group). For investigation of the relevant gene changes in tissues, two mice in each group as control were chosen escaping the fatigue test.

### Tissue collection

Animals were euthanized 72 h after the last bout of exercise. Gastrocnemius, quadriceps and heart muscles were thus isolated, frozen, and stored at −80°C until further analysis.

### Statistical analysis

All data were reported as mean ± standard deviation of the mean (SD). Data were analyzed in either one-way ANOVA with an appropriate post hoc test for comparison of multiple groups or unpaired student's t test for comparison of two groups as described in figure legends (Graphpad Prism software).

## Supporting Information

Figure S1 **Arctigenin (ATG) enhanced AMPK phosphorylation in HEK293T cells.** HEK293T cells were incubated with indicated concentrations of arctigenin (0-40 µM) for 30 min, phospho- and total AMPK were then detected by western blotting. The results shown are representative of three independent experiments. The bands were quantified using Image-Pro Plus software. Values are means ± SE. **, p<0.05; ***, p<0.005; one-way ANOVA.
(TIF)

Figure S2 **Arctigenin (ATG) activated PGC-1α transcription via up-regulating AMPK phosphorylation.** **A**. When the confluence reached 30∼40% (24-well plate), HEK293T cells were transiently transfected with pGL3-PGC-1α promoter-Luc and SV40. 5 hours later, cells were refreshed with medium supplemented with arctigenin (1, 10, 40 µM) or DMSO and incubated for 24 hours before Luciferase assays as described in “Materials and methods”. **B**. After transfection, HEK293T cells were administrated with or without 20 µM compound C for 1 hour before and during the incubation with actigenin (40 µM) for 24 hours before Luciferase assays as described in “Materials and methods”. **, p<0.01. ##, p<0.01: for compound C and arctigenin co-incubation group versus arctigenin treated group; student's t test.
(TIF)

Figure S3 **Effects of arctigenin (ATG) on ERRα, cytochrome c, PDK4, SCD1, FAS and mCPT1b were subjective to AMPK phosphorylation in H9C2.** H9C2 cells were treated with or without 20 µM compound C for 1 hour before and during the incubation with actigenin (20 µM) for 24 hours. After harvested, mRNA levels of ERRα (A), cytochrome c (B), SCD1 (C), PDK4 (D), FAS (E) and mCPT1b (F) were analyzed. The results shown are representative of three independent experiments. Values are means ± SD. *, p<0.05. #, p<0.05: for compound C and arctigenin co-incubation group versus arctigenin treated group; student's t test.
(TIF)

Figure S4 **Effects of arctigenin (ATG) on ERRα, cytochrome c, PDK4, SCD1, FAS and mCPT1b were subjective to AMPK phosphorylation in C2C12.** Differentiated C2C12 cells were administrated with or without 20 µM compound C for 1 hour before and during the incubation with actigenin (20 µM) for 24 hours. After harvested, mRNA levels of ERRα (A), cytochrome c (B), SCD1 (C), PDK4 (D), FAS (E) and mCPT1b (F) were analyzed. The results shown are representative of three independent experiments. Values are means ± SD. *, p<0.05; **, p<0.01; ***, p<0.005. #, p<0.05; ##, p<0.01; ###, p<0.005: for compound C and arctigenin co-incubation group versus arctigenin treated group; student's t test.
(TIF)

Figure S5 **Arctigenin (ATG) failed to regulate the co-activator recruitment and transcriptional activity of PPARδ.** **A**. HEK293T cells were transfected with UAS-TK-Luc, pCMX-Gal4DBD-PPARδ-LBD and pRL-SV40 followed by treatment of DMSO, GW501516 (PPARδ agonist), and varied concentrations of arctigenin for 24 hours. **B**. HEK293T cells were transfected with pAdTrack-PPARδ, pcDNA3.1-RXRα, pSV-PPRE-Luc and pRL-SV40 and then incubated with DMSO, GW501516 (PPARδ agonist), and varied concentrations of arctigenin for 24 hours. **C**. HEK293T cells were transfected with pSV-PPRE-Luc and pRL-SV40, and incubated with DMSO, GW501516 (PPARd agonist), and varied concentrations of arctigenin for 24 hours. Relative luciferase activities were measured as described in Text S1. The results shown are representative of three independent experiments. Values are means ± SD. *, p<0.05; **, p<0.01; ***, p<0.005; one-way ANOVA.
(TIF)

Figure S6 **Effects of arctigenin (ATG) on diet, weight, inflammation and liver toxicity of mice.** **A**. Daily food intake of each group was analyzed (n = 10/group). **B**. Weight change in each group was measured (n = 10/group). **C**. **D**. Serum from mice in each group was collected and levels of TNFα (C) and IL-6 (D) were analyzed (n = 10/group). **E**. **F**. Activities of ALT (E) and AST (F) were measured (n = 10/group). Values are means ± SE. *, p<0.5; **, p<0.01; student's t test.
(TIF)

Figure S7 **Arctigenin (ATG) failed to induce skeletal muscle fiber-type change.** Metachromatical staining of frozen cross-sections from gastrocnemius and quadriceps in vehicle and arctigenin treated groups. The results shown are representative of three independent experiments. Dark-brown stained type I fibers were indicated by arrows.
(TIF)

Figure S8 **Arctigenin (ATG) enhanced fatty acid storage in gastrocnemius.** Free fatty acid in gastrocnemius (A) or quadriceps (B) of each group was analyzed (n = 7/group). Values are means ± SD. *, p<0.5; student's t test.
(TIF)

Figure S9 **Arctigenin (ATG) failed to impact the phosphorylation of AMPK on Ser485/491 sites.** HEK293T, H9C2 and differentiated C2C12 cells were incubated with indicated concentrations of arctigenin (0–40 µM) for 30 min, AMPK (Thr172), AMPK (Ser485/491) and total AMPK were then detected by western blotting. The results shown are representative of three independent experiments.
(TIF)

Figure S10 **A scheme demonstrating recombinant AMPK activity assay approach.**
(TIF)

Text S1 **Supporting documents.**
(DOC)
