# Supplementary figures and images for: Arctigenin Efficiently Enhanced Sedentary Mice Treadmill Endurance
Source: PLoS One. 2011 Aug 26;6(8):e24224. doi: 10.1371/journal.pone.0024224 (PMC3162610; doi:10.1371/journal.pone.0024224)

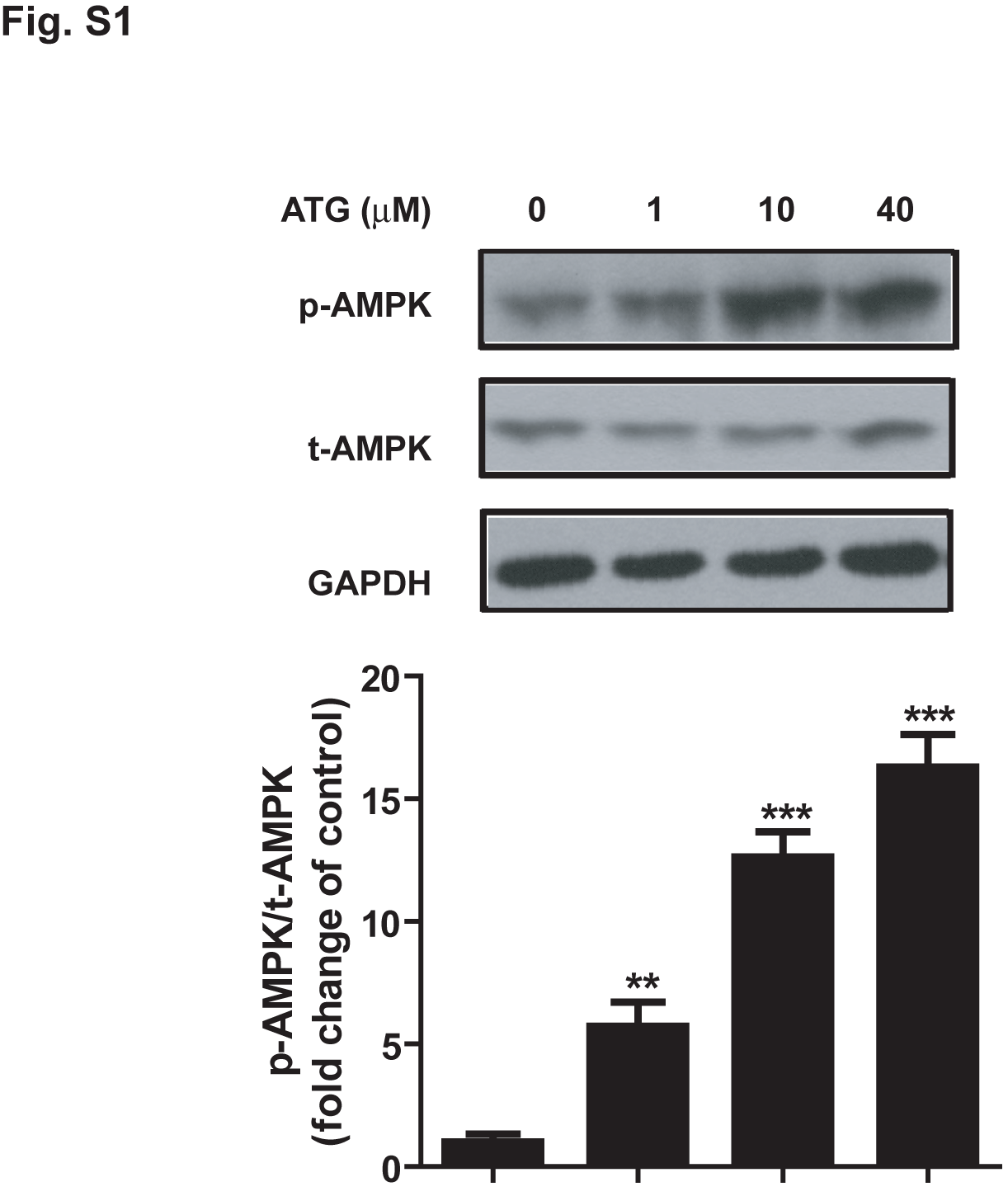

Supplement: Figure S1 — Arctigenin (ATG) enhanced AMPK phosphorylation in HEK293T cells. HEK293T cells were incubated with indicated concentrations of arctigenin (0-40 µM) for 30 min, phospho- and total AMPK were then detected by western blotting. The results shown are representative of three independent experiments. The bands were quantified using Image-Pro Plus software. Values are means ± SE. **, p<0.05; ***, p<0.005; one-way ANOVA. (TIF) [file pone.0024224.s001.tif]

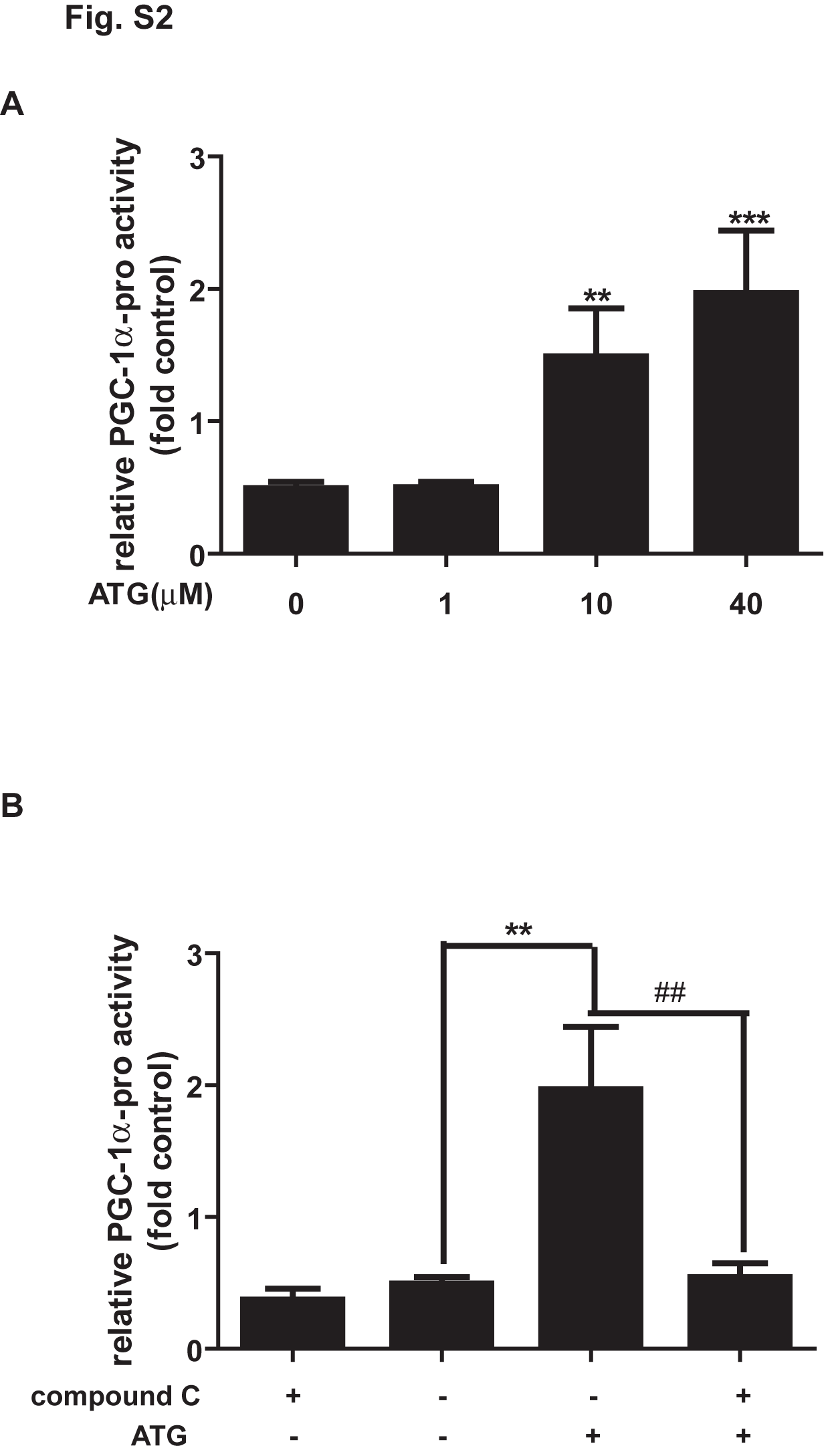

Supplement: Figure S2 — Arctigenin (ATG) activated PGC-1α transcription via up-regulating AMPK phosphorylation. A. When the confluence reached 30∼40% (24-well plate), HEK293T cells were transiently transfected with pGL3-PGC-1α promoter-Luc and SV40. 5 hours later, cells were refreshed with medium supplemented with arctigenin (1, 10, 40 µM) or DMSO and incubated for 24 hours before Luciferase assays as described in “Materials and methods”. B. After transfection, HEK293T cells were administrated with or without 20 µM compound C for 1 hour before and during the incubation with actigenin (40 µM) for 24 hours before Luciferase assays as described in “Materials and methods”. **, p<0.01. ##, p<0.01: for compound C and arctigenin co-incubation group versus arctigenin treated group; student's t test. (TIF) [file pone.0024224.s002.tif]

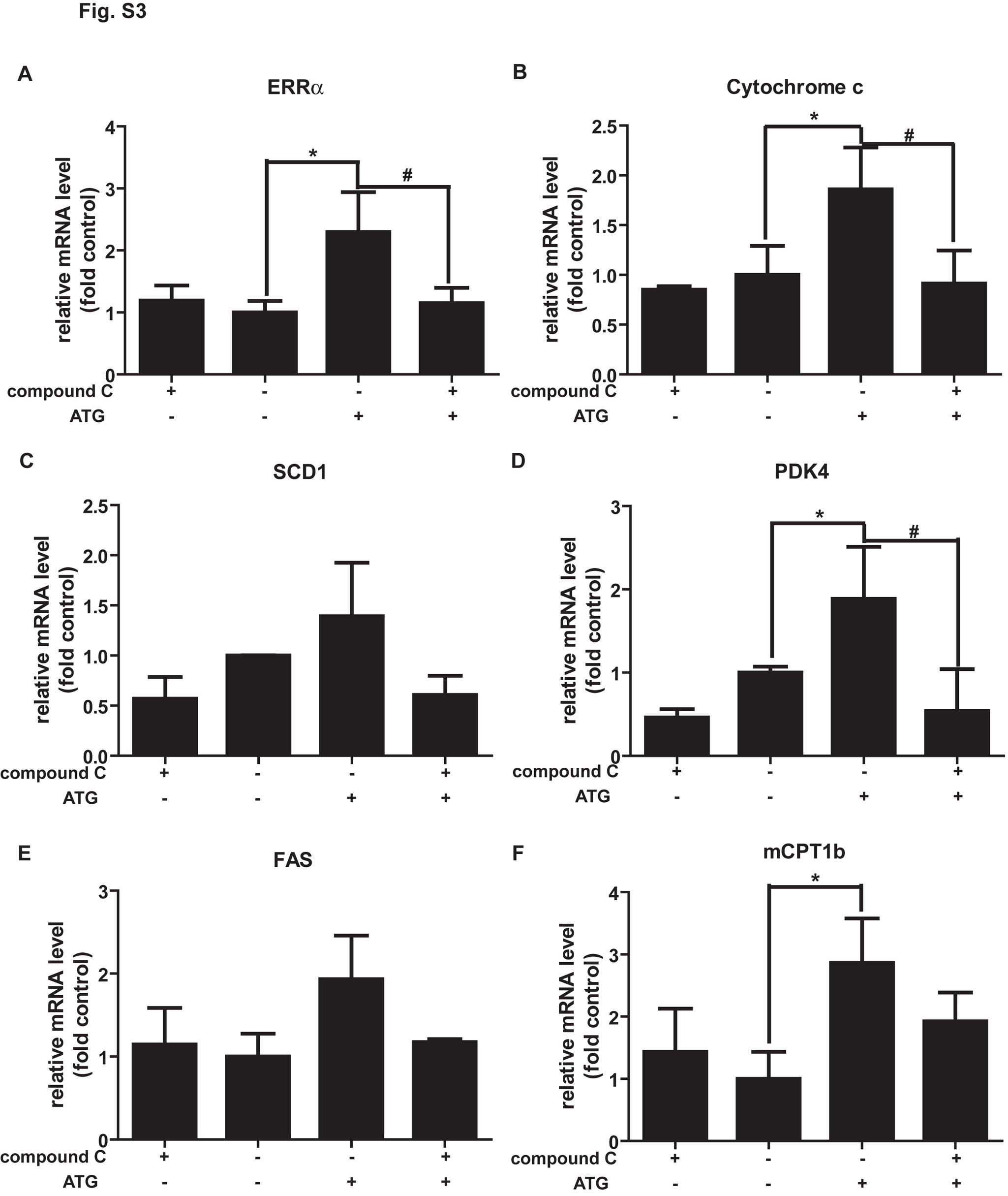

Supplement: Figure S3 — Effects of arctigenin (ATG) on ERRα, cytochrome c, PDK4, SCD1, FAS and mCPT1b were subjective to AMPK phosphorylation in H9C2. H9C2 cells were treated with or without 20 µM compound C for 1 hour before and during the incubation with actigenin (20 µM) for 24 hours. After harvested, mRNA levels of ERRα (A), cytochrome c (B), SCD1 (C), PDK4 (D), FAS (E) and mCPT1b (F) were analyzed. The results shown are representative of three independent experiments. Values are means ± SD. *, p<0.05. #, p<0.05: for compound C and arctigenin co-incubation group versus arctigenin treated group; student's t test. (TIF) [file pone.0024224.s003.tif]

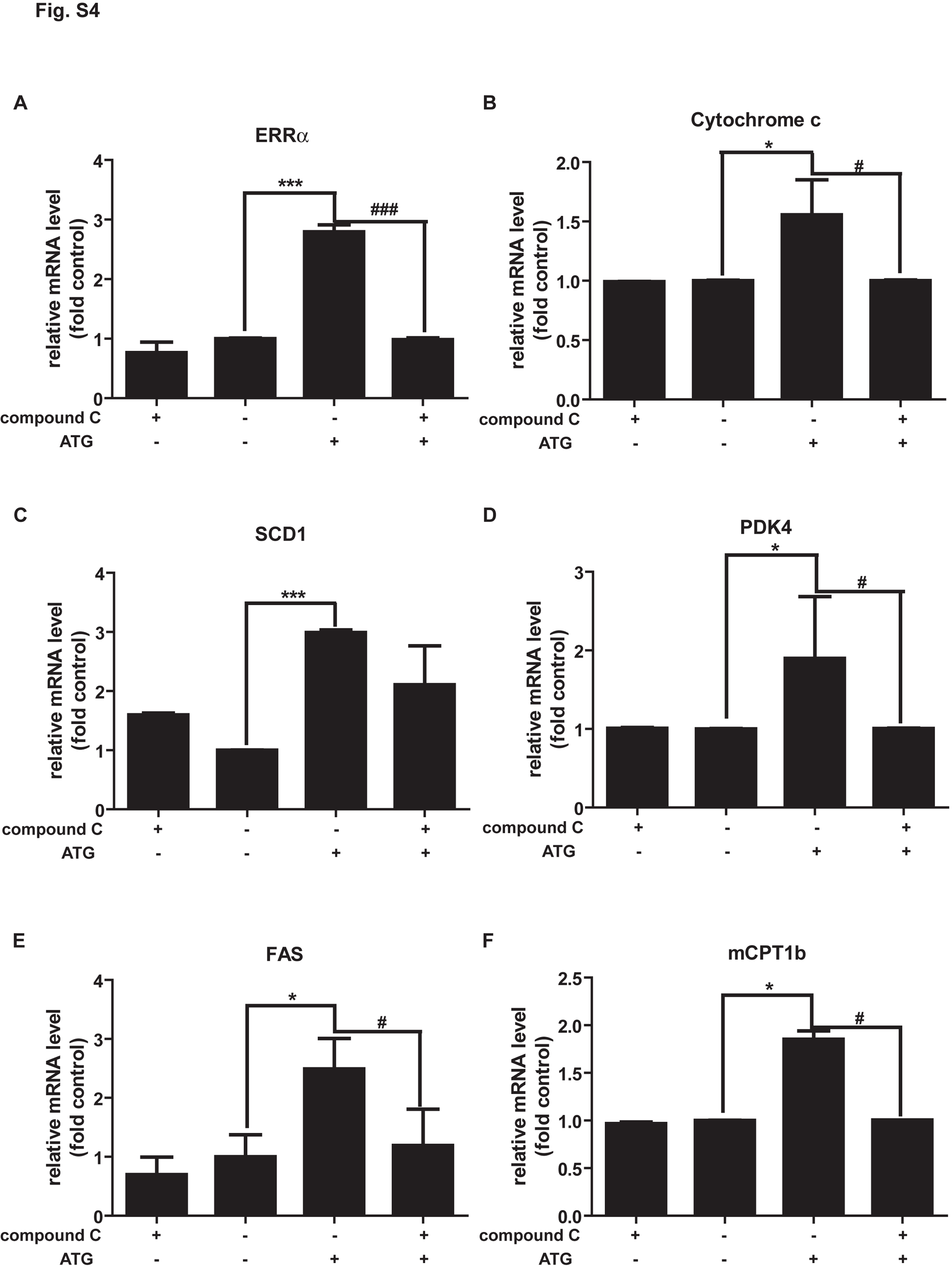

Supplement: Figure S4 — Effects of arctigenin (ATG) on ERRα, cytochrome c, PDK4, SCD1, FAS and mCPT1b were subjective to AMPK phosphorylation in C2C12. Differentiated C2C12 cells were administrated with or without 20 µM compound C for 1 hour before and during the incubation with actigenin (20 µM) for 24 hours. After harvested, mRNA levels of ERRα (A), cytochrome c (B), SCD1 (C), PDK4 (D), FAS (E) and mCPT1b (F) were analyzed. The results shown are representative of three independent experiments. Values are means ± SD. *, p<0.05; **, p<0.01; ***, p<0.005. #, p<0.05; ##, p<0.01; ###, p<0.005: for compound C and arctigenin co-incubation group versus arctigenin treated group; student's t test. (TIF) [file pone.0024224.s004.tif]

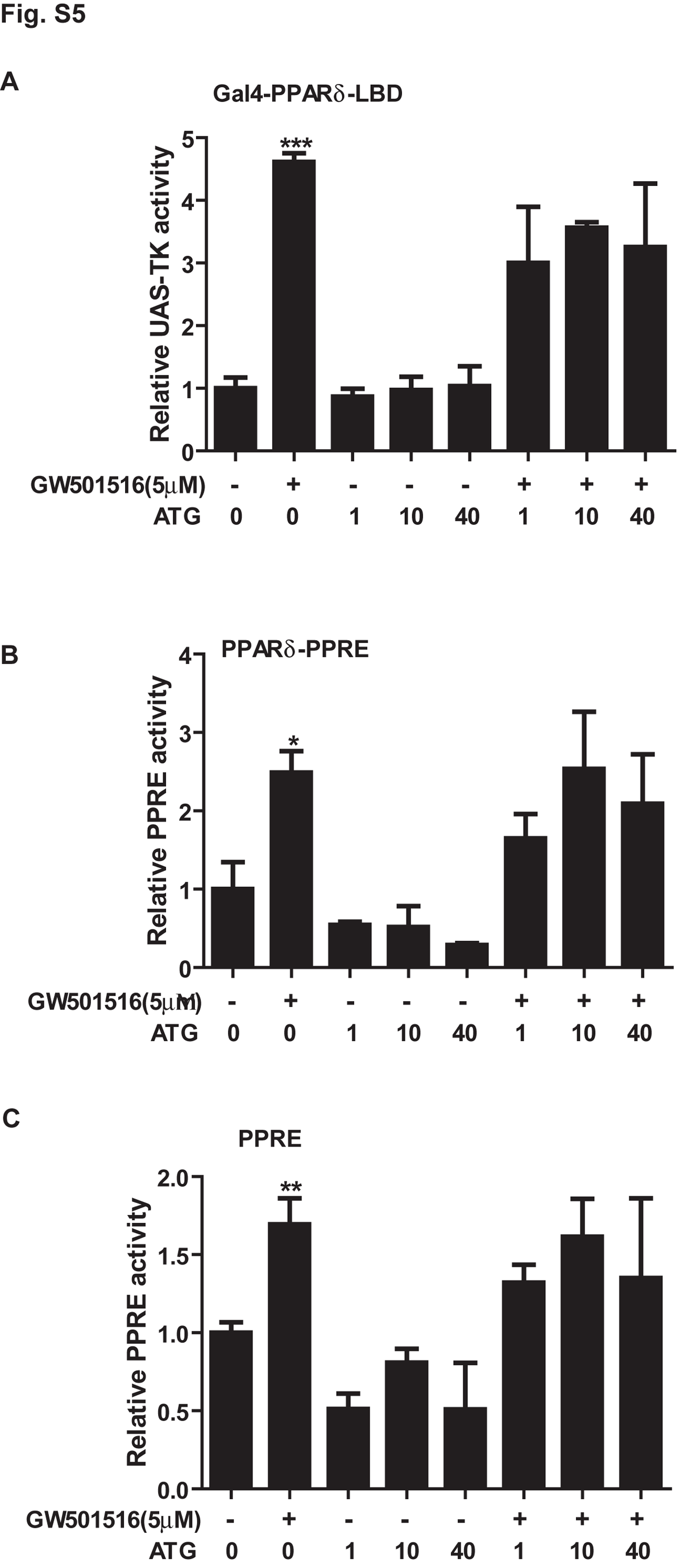

Supplement: Figure S5 — Arctigenin (ATG) failed to regulate the co-activator recruitment and transcriptional activity of PPARδ. A. HEK293T cells were transfected with UAS-TK-Luc, pCMX-Gal4DBD-PPARδ-LBD and pRL-SV40 followed by treatment of DMSO, GW501516 (PPARδ agonist), and varied concentrations of arctigenin for 24 hours. B. HEK293T cells were transfected with pAdTrack-PPARδ, pcDNA3.1-RXRα, pSV-PPRE-Luc and pRL-SV40 and then incubated with DMSO, GW501516 (PPARδ agonist), and varied concentrations of arctigenin for 24 hours. C. HEK293T cells were transfected with pSV-PPRE-Luc and pRL-SV40, and incubated with DMSO, GW501516 (PPARd agonist), and varied concentrations of arctigenin for 24 hours. Relative luciferase activities were measured as described in Text S1. The results shown are representative of three independent experiments. Values are means ± SD. *, p<0.05; **, p<0.01; ***, p<0.005; one-way ANOVA. (TIF) [file pone.0024224.s005.tif]

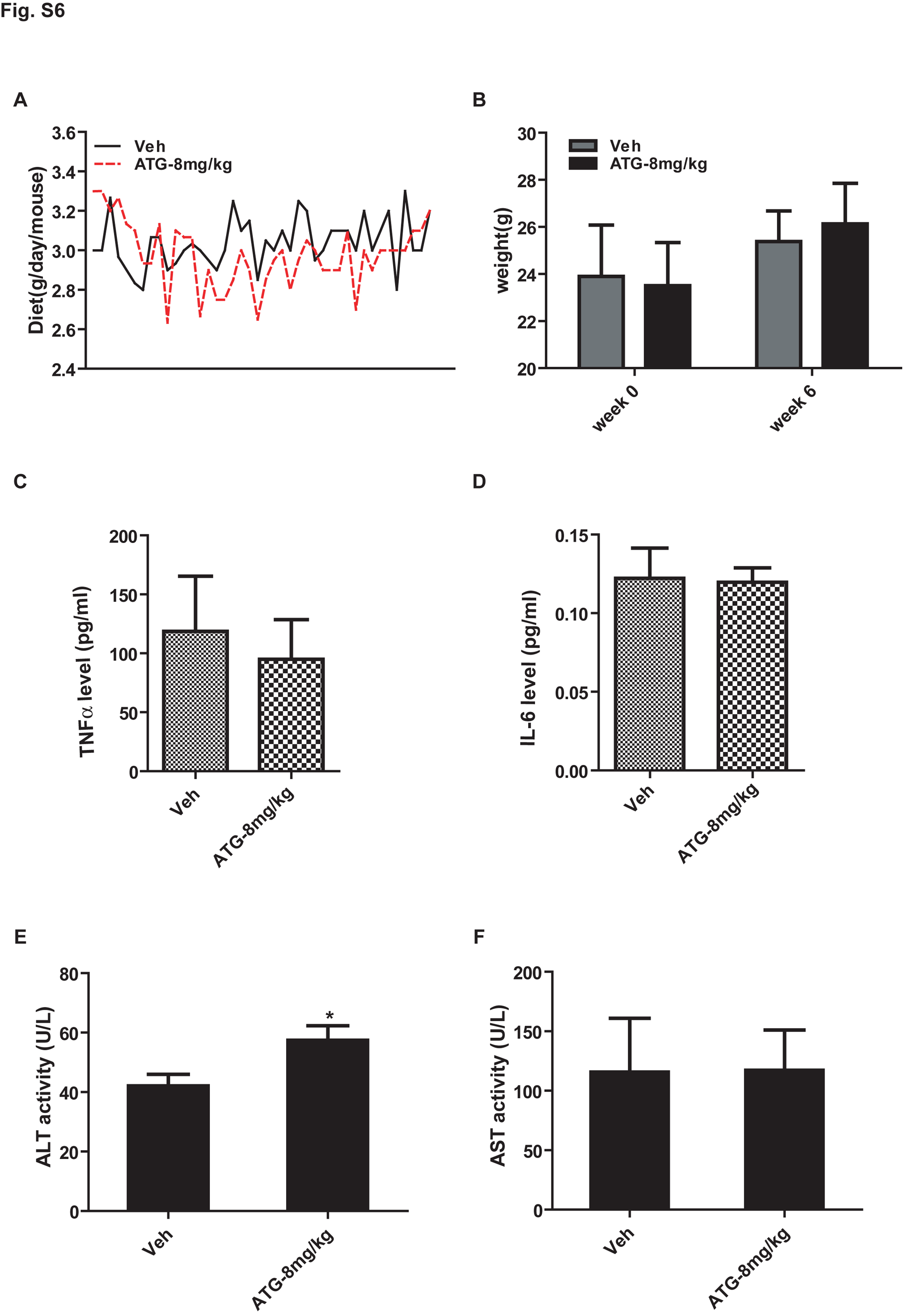

Supplement: Figure S6 — Effects of arctigenin (ATG) on diet, weight, inflammation and liver toxicity of mice. A. Daily food intake of each group was analyzed (n = 10/group). B. Weight change in each group was measured (n = 10/group). C. D. Serum from mice in each group was collected and levels of TNFα (C) and IL-6 (D) were analyzed (n = 10/group). E. F. Activities of ALT (E) and AST (F) were measured (n = 10/group). Values are means ± SE. *, p<0.5; **, p<0.01; student's t test. (TIF) [file pone.0024224.s006.tif]

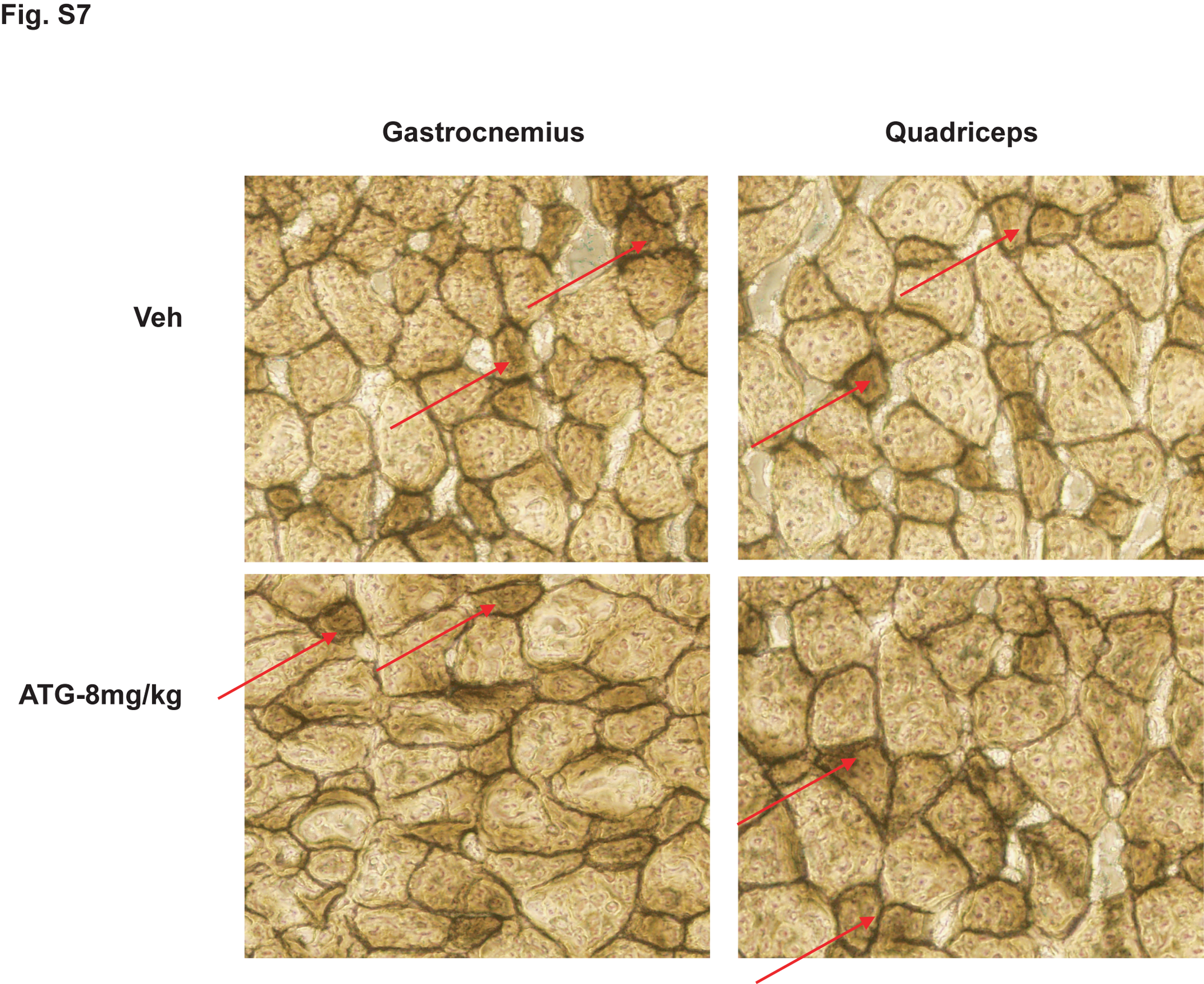

Supplement: Figure S7 — Arctigenin (ATG) failed to induce skeletal muscle fiber-type change. Metachromatical staining of frozen cross-sections from gastrocnemius and quadriceps in vehicle and arctigenin treated groups. The results shown are representative of three independent experiments. Dark-brown stained type I fibers were indicated by arrows. (TIF) [file pone.0024224.s007.tif]

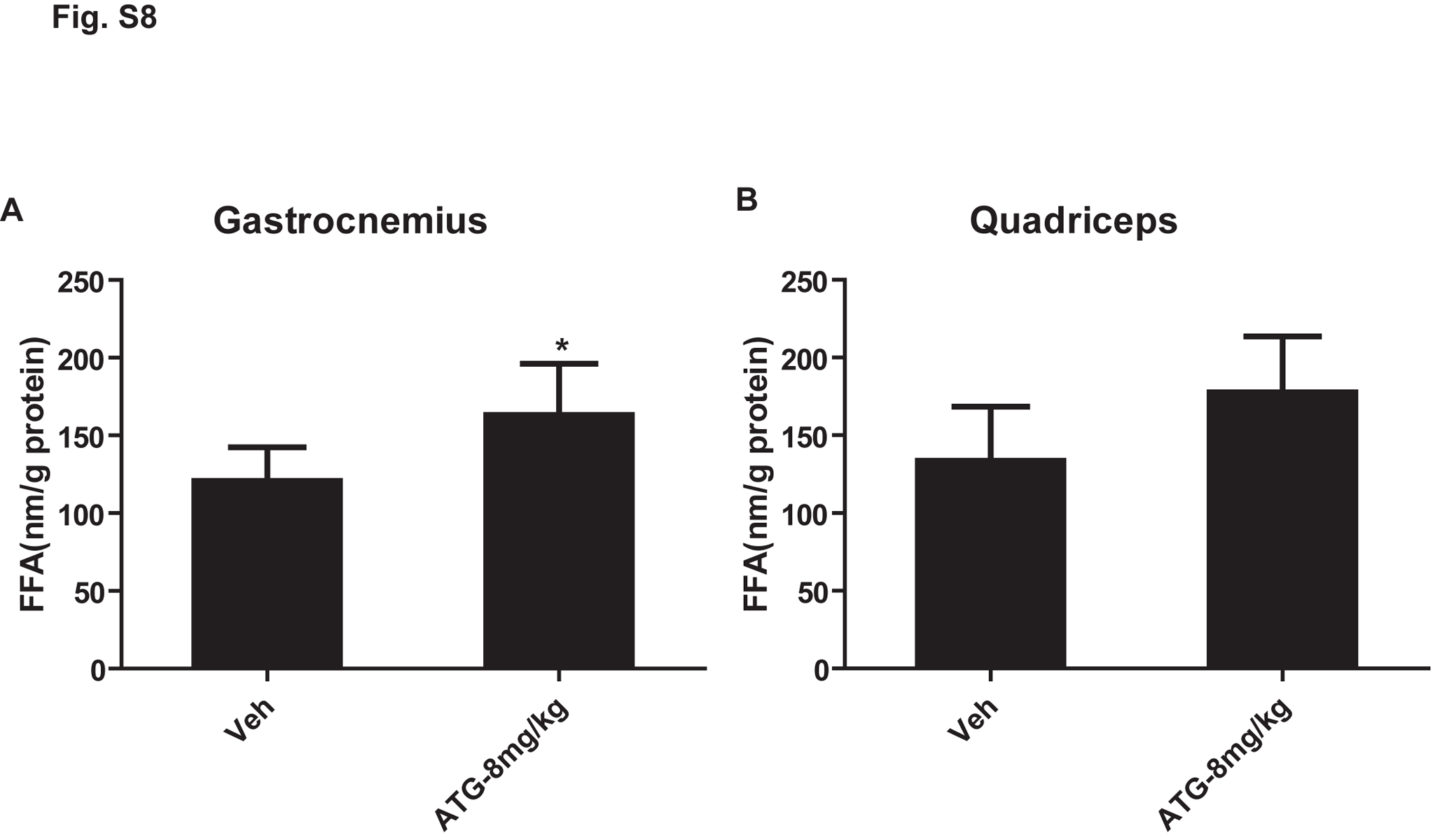

Supplement: Figure S8 — Arctigenin (ATG) enhanced fatty acid storage in gastrocnemius. Free fatty acid in gastrocnemius (A) or quadriceps (B) of each group was analyzed (n = 7/group). Values are means ± SD. *, p<0.5; student's t test. (TIF) [file pone.0024224.s008.tif]

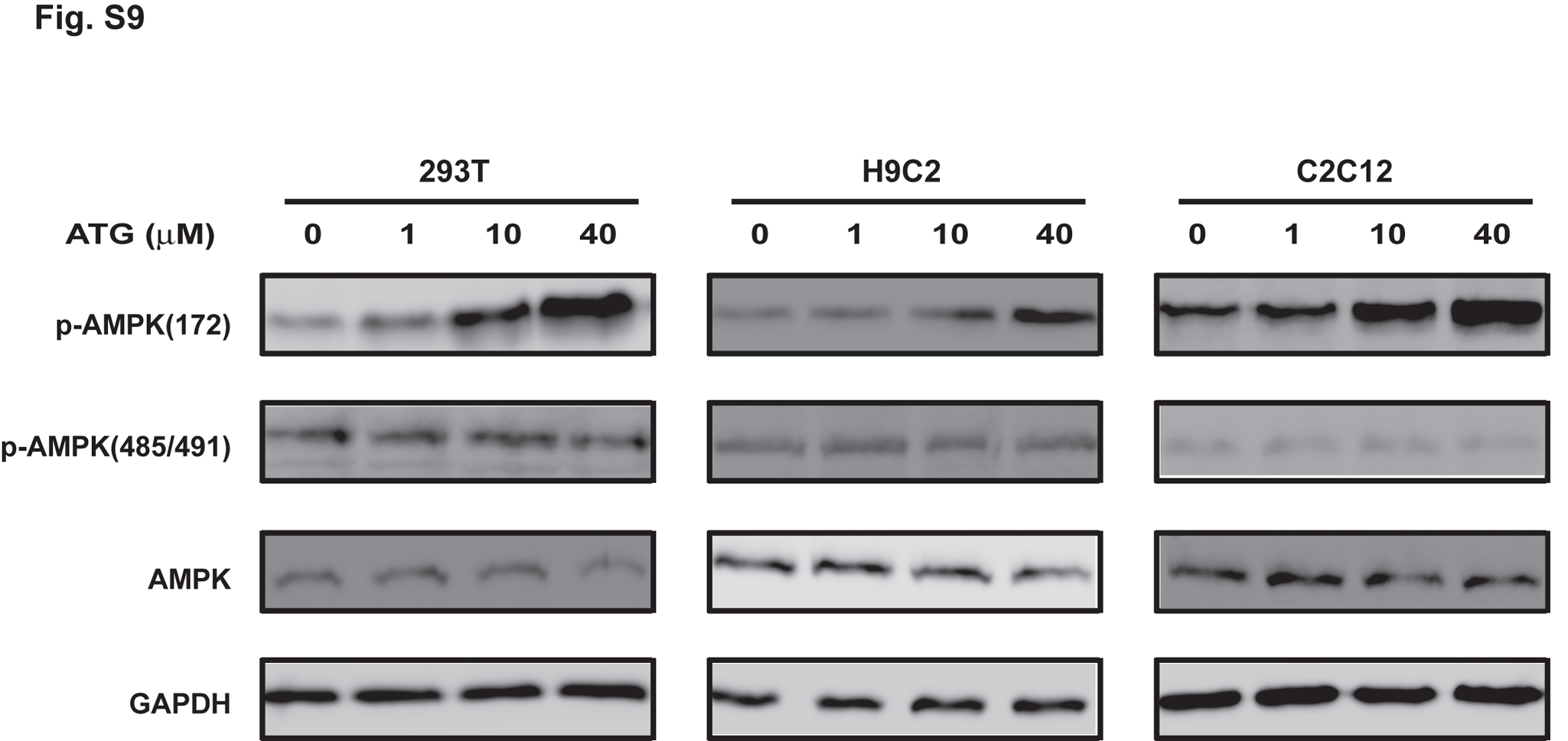

Supplement: Figure S9 — Arctigenin (ATG) failed to impact the phosphorylation of AMPK on Ser485/491 sites. HEK293T, H9C2 and differentiated C2C12 cells were incubated with indicated concentrations of arctigenin (0–40 µM) for 30 min, AMPK (Thr172), AMPK (Ser485/491) and total AMPK were then detected by western blotting. The results shown are representative of three independent experiments. (TIF) [file pone.0024224.s009.tif]

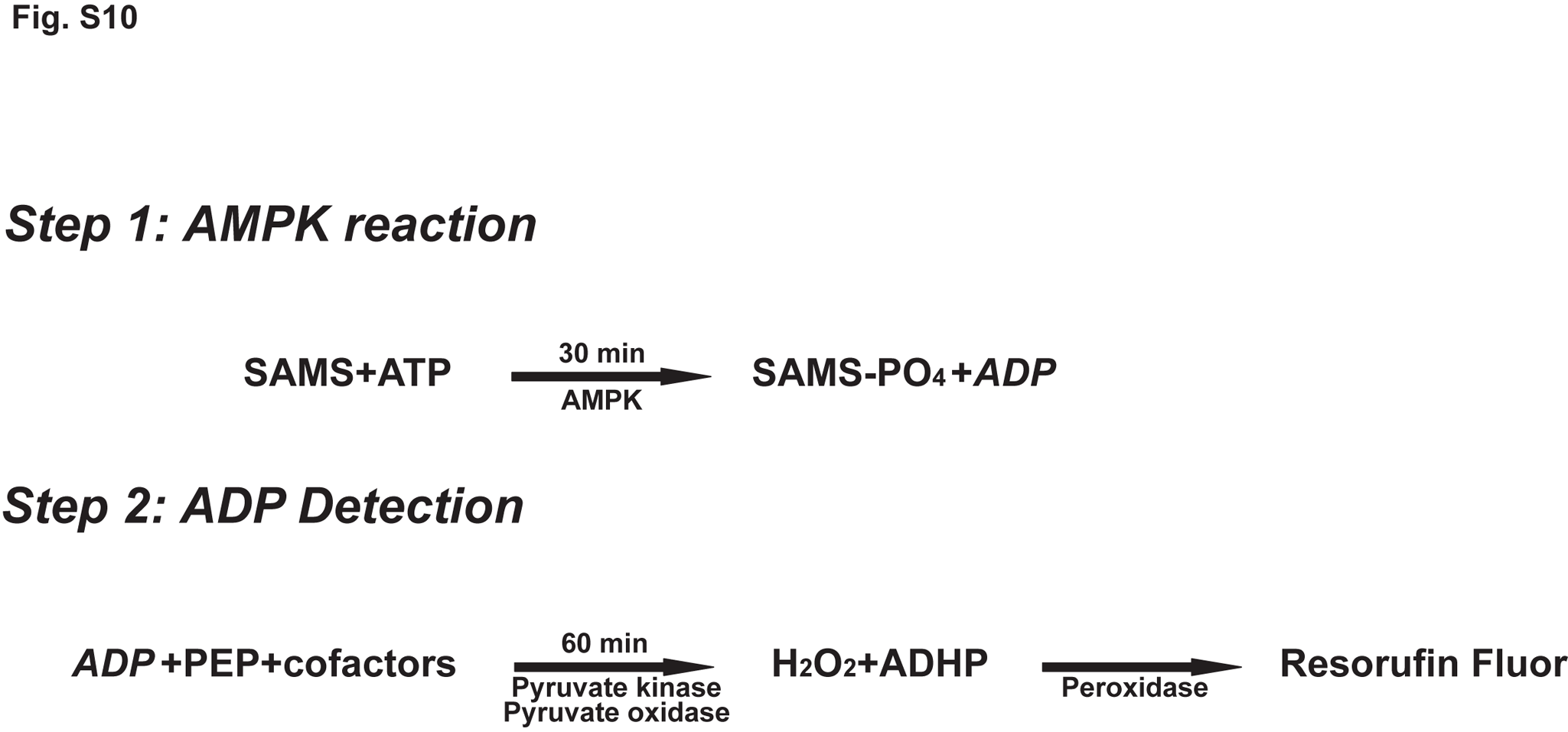

Supplement: Figure S10 — A scheme demonstrating recombinant AMPK activity assay approach. (TIF) [file pone.0024224.s010.tif]
