# Supplementary material for: Arctigenin Efficiently Enhanced Sedentary Mice Treadmill Endurance
Source: PLoS One. 2011 Aug 26;6(8):e24224. doi: 10.1371/journal.pone.0024224 (PMC3162610; doi:10.1371/journal.pone.0024224)
Supplement: Text S1 — Supporting documents. (DOC) [file pone.0024224.s011.doc]

**Supporting documents**

**Arctigenin efficiently enhanced sedentary mice treadmill endurance**

Xuan Tang^1,#^, Jingjing Zhuang^2,#^, Jing Chen^1^,*, Liang Yu^1^, Lihong Hu^1^,*, Hualiang Jiang^1^, Xu Shen^1,2^,*

^1^State Key Laboratory of Drug Research, Shanghai Institute of Materia Medica, Chinese Academy of Sciences, Shanghai 201203, China. ^2^School of Pharmacy, East China University of Science and Technology, Shanghai 200237, China.

^#^These two authors contributed equally to this work.

*Corresponding authors.

Mailing address: Shanghai Institute of Materia Medica, Chinese Academy of Sciences, Shanghai 201203, China.

Tel&Fax: +86-21-50806918.

E-mail: jingchen@mail.shcnc.ac.cn (J. Chen), xshen@mail.shcnc.ac.cn (X. Shen) and simmhulh@mail.shcnc.ac.cn (L. Hu)

**Materials and methods**

**Materials**

GW501516 was purchased from Applichem. Sodium pentobarbital, calcium chloride, cobalt chloride, ammonium sulfide were analytical pure and purchased from Sinopharm Chemical Reagent Co., Ltd. ATP disodium was obtained from Sangon Biotech Co., Ltd. Anti-phospho-AMPK (Ser485/491) was purchased from Cell Signaling Technology. UAS-TK-Luc reporter was generously donated by Dr. Daniel P. Kelly (Washington University School of Medicine, USA). pSV-PPRE-Luc reporter was described previously [1]. pcDNA3.1-RXRα was kindly provided by Dr. Gordon Hager (National Cancer Institute, Laboratory of Receptor Biology and Gene Expression). The fusion construct of pCMX-Gal4DBD-PPARδ-LBD was generated by using pAdTrack-PPARδ (generally provided by Dr B VOGELSTEIN, Howard Hughes Medical Institute, USA) as template. Mouse IL-6 Elisa kit and Mouse TNF-α Elisa kit were purchased from RB.

**p-AMPK activator screening platform**

For screening AMPK activators, HEK293T cells were incubated with indicated concentrations of compound for 30 min. AMPK phosphorylation level was then detected by western blot to evaluate the compound effect.

**Metachromatical ATPase staining**

Frozen gastrocnemius and quadriceps tissues were cross-sectioned in 10μm sections. ATPase staining was performed according to a modified protocol [2]. The sections were incubated in alkaline preincubating solution (0.02 mol/L sodium pentobarbital, 0.036 mol/L calcium chloride) at pH 10.0 for 15 min and then in substrate solution (0.02 mol/L sodium pentobarbital, 0.018 mol/L calcium chloride, 2.5 g/L ATP disodium) at pH 9.4 for 45 min. The sections were washed by 1% (v/v) calcium chloride for 1 min three times followed by incubation in 2% (v/v) cobalt chloride for 3 min. The sections were subsequently washed by 0.01 mol/L sodium pentobarbital five times and immersed in 2% (v/v) ammonium sulfide for 30 sec.

**Determination of tissue FFA**

Gastrocnemius and quadriceps FFA detections were performed according to the Kit instruction of ultrasensitive assay kit for free fatty acids (Applygen Technologies Inc. Beijing).

**Determination of serum TNFα and IL-6 levels**

Blood samples were obtained at time of sacrifice. Fresh blood samples were centrifuged at 3000 rpm for 10 min at 4 °C. Serum supernatant was collected and frozen at -80 °C for later analysis. IL-6 and TNF-α levels were determined according to the related Elisa kit instruction.

**Determination of serum AST and ALT activities**

Serum aspartate aminotransferase (AST) and alanine aminotransferase (ALT) were detected by a commercially available kit with a HITACHI 7020 autoanalyzer (Hitachi Inc., Hapen).

**Mammalian one-hybrid assay**

HEK293T cells were transiently transfected with the UAS-TK-Luc reporter, the fusion construct of pCMX-Gal4DBD-PPARδ-LBD and pRL-SV40, and then treated with compounds or DMSO after 5-h transfection. Cells were harvested, and luciferase activities were analyzed 24 hours later.

**PPRE transactivation assay**

PPRE transcriptional activation assay was performed to investigate the potential effects of arctigenin on PPARδ related transcriptional activity. HEK293T cells were co-transfected with pAdTrack-PPARδ, pcDNA3.1-RXRα, pSV-PPRE-Luc and pRL-SV40 or pSV-PPRE-Luc and pRL-SV40. After 5-h transfection, the cells were incubated with compounds or DMSO for further 24 hours and then lysed, and luciferase activities were measured.

**References**

1. Zou G, Gao Z, Wang J, Zhang Y, Ding H et al (2008) Deoxyelephantopin inhibits cancer cell proliferation and functions as a selective partial agonist PPARgamma. Biochem Pharmacol 75:1381-1392.

2. Qiu SD, Song TB (2008) Histochemistry and Immunohistochemistry. Beijing: Science Press. 91-92 p.
